# Challenging a Myth and Misconception: Red-Light Vision in Rats

**DOI:** 10.3390/ani10030422

**Published:** 2020-03-03

**Authors:** Stephanie Niklaus, Silvio Albertini, Tobias K. Schnitzer, Nora Denk

**Affiliations:** Pharma Research and Early Development (pRED), Pharmaceutical Sciences (PS), Roche Innovation Center Basel, 4070 Basel, Switzerland; stephanie.niklaus@roche.com (S.N.); silvio.albertini@roche.com (S.A.); tobias.schnitzer@roche.com (T.K.S.)

**Keywords:** electroretinogram, rat, red light, husbandry, photoreceptors, rods, cones, retina

## Abstract

**Simple Summary:**

Light substantially influences animal physiology and behavior. Thus, it is a prerequisite to house laboratory animals under optimal light conditions. Different species possess different sets of photoreceptors, resulting in differential perception of the visible-light spectrum. While humans are trichromats with red-, green- and blue-sensitive cones, rats and mice are dichromats possessing ultraviolet- and green-sensitive cones. This led to the common assumption that red light is invisible to rodents and therefore red lights are commonly used in husbandry and experiments to observe animals during their dark phase. The retinal sensitivity of rats to red light though has never been assessed under scotopic conditions (dark-adapted) even though this mimics the setting red observation lights are being used. We examined the sensitivity to far-red light of the dark- and light-adapted rat retina. Our study demonstrates that the rat retina responds to far-red light under both conditions with great sensitivity, indicating that rats are not red-light blind. This should be taken into consideration when using red light to keep the effects of light on the retina and physiology to a minimum and will improve animal well-being and lead to better quality data by decreasing the variable light.

**Abstract:**

Due to the lack of L-cones in the rodent retina, it is generally assumed that red light is invisible to rodents. Thus, red lights and red filter foils are widely used in rodent husbandry and experimentation allowing researchers to observe animals in an environment that is thought to appear dark to the animals. To better understand red-light vision in rodents, we assessed retinal sensitivity of pigmented and albino rats to far-red light by electroretinogram. We examined the sensitivity to red light not only on the light- but also dark-adapted retina, as red observation lights in husbandry are used during the dark phase of the light cycle. Intriguingly, both rods and cones of pigmented as well as albino rats show a retinal response to red light, with a high sensitivity of the dark-adapted retina and large electroretinogram responses in the mesopic range. Our results challenge the misconception of rodents being red-light blind. Researchers and housing facilities should rethink the use of red observation lights at night.

## 1. Introduction

Light substantially affects an animal’s appearance, behavior and physiology, far beyond its effects on the eye. Being the most important zeitgeber of the circadian rhythm, light influences an animal’s diurnality, endocrine levels, and metabolism [1,2]. Furthermore, depending on level of illuminance, spectrum, and photoperiod, light has the potential to damage eyes and skin [3]. Given the tremendous impact of light on organisms, it is essential to ensure optimal illumination in laboratory animal facilities to maximize animal well-being.

Vision is rendered in the retina. Photoreceptors convert the physical light stimulus into an electrical stimulus, which, via retinal interneurons, is further transmitted to the brain. Rats and mice are the most frequently used laboratory mammals, hence understanding exact species- and strain-specific differences in visual processing, including the range of spectral sensitivity, is key not only for ophthalmic studies but also to ensure appropriate environmental lighting conditions for husbandry. Rats and mice, being nocturnal animals, possess a rod-dominant retina with a relative percentage of cones as low as 1% and 3%, respectively [4,5,6]. Rats and mice have dichromatic vision, in contrast to the trichromatic vision of humans. The two sets of rodent cones exhibit a peak sensitivity in the ultraviolet (UV) range (UV cones; peak absorption at 358–359 nm and 360 nm for rats and mice, respectively) [7,8,9] and in the green spectrum (M cones; peak absorption at 509–510 nm and 508–511 nm for rats and mice, respectively) [4,9,10,11,12]. Retinal function is typically assessed by electroretinography (ERG), where the sum field potential changes evoked by light stimuli are measured, which consist of a negative a-wave, followed by a positive b-wave (representing hyperpolarization and depolarization of photoreceptors and ON-bipolar cells, respectively). Photopic spectral sensitivity curves of image-forming photoreceptors of rats and mice have recently been determined by a constant response ERG, where response amplitudes were kept constant by adjusting light intensities throughout the UV-visible spectrum (300 to 700 nm) [13]. These measurements revealed two sensitivity peaks in rats at 362 and 502 nm, corresponding to the peak absorption wavelengths of their UV- and M-cones [13]. No significant responses to long wavelength light (above 620 nm) were detected [13]. In addition to rods and cones, light is also perceived by the non-image forming intrinsically photosensitive retinal ganglion cells (ipRGCs) located in the inner retina. These ipRGCs possess the photopigment melanopsin, which is sensitive to blue/green light with a peak absorption at 480 nm and an insensitivity to red light (above 620 nm) [14,15,16,17]. Image-forming photoreceptors and ipRGCs play complimentary roles in the entrainment of the circadian system (reviewed by Ketema et al., 2009) [18].

The characterization of the photopic spectral sensitivity of rats and mice [13] supported the misconception of red-light blindness in rodents, which had long been in place based on the inability to identify red-light sensitive cones. In combination with the insensitivity of melanopsin expressing ipRGCs to long wavelengths, red light was assumed to have no influence on the retinal dark adaptation state, nor on an animal’s physiology and behavior. Based on the general assumption that red light is being perceived as darkness by rodents, red light was, and still is, commonly used in reverse light cycle conditions and when working with dark-adapted animals in ophthalmic studies.

Nevertheless, there is evidence coming from different studies contradicting this assumption. Short durations of high-intensity red-light exposure during the dark phase suppresses melatonin synthesis in rats to a similar extent as white light [19,20]. Furthermore, even dim-red light during the dark phase is sufficient to alter the circadian rhythm, measured by locomotor activity and sleep-wake behavior in mice [21,22]. Additionally, red light is sufficient to entrain the circadian oscillations of body temperature, though higher intensities are required compared to green light [23]. Exposure of female rats to continuous red light at a peak wavelength of 660 nm is sufficient to induce persistent estrus [24]. Moreover, a thorough study on the influence of low intensity red light at night on circadian metabolism and physiology in rats revealed decreased plasma melatonin levels, a lack of rhythmic changes in the total fatty acid content in the plasma, higher arterial levels of glucose and lactate, altered corticosterone rhythmicity, and alterations in the concentrations of leptin and insulin and the duration of their rhythms [25]. Whether these effects of red light on the circadian rhythm are mediated by ipRGCs, rods or cones or by a combination of all three types of photoreceptors, remains open, but highlights the necessity of investigating the possibility of far red-light vision in rats and the potential mechanisms through which these changes occur.

Therefore, we assessed retinal responses mediated by rods and cones of pigmented (Brown Norway) and albino (Wistar) rats in response to monochromatic far red light of 656 +/−10 nm. Besides the assessment under photopic (light-adapted) condition, we included recordings in a dark-adapted (scotopic) setting to examine the impact of red observation lights used during the dark phase in husbandry and during experiments. Both rat strains showed a significant scotopic and photopic ERG response to red light even at low intensities. Red-light response amplitudes were smaller than those evoked by white light, however mesopic red-light responses were surprisingly high, emphasizing that even far-red light is, contrary to the common assumption, very well perceived by rats.

## 2. Materials and Methods

### 2.1. Animals and Husbandry

All animal experiments were conducted with the approval of the cantonal (BS) Swiss veterinary authorities and are in accordance with the Swiss Animal Welfare Act (TSchG), Art. 141 of the Animal Welfare Ordinance (TSchV), and Art. 30 of the Animal Experimentation Ordinance (TVV).

Ten pigmented rats of the Brown Norway strain (5 female and 5 male) and 10 albino rats of the Wistar strain (5 female and 5 male) were used for this study. Rats were group-housed (2 to 3 animals per cage) at 22+/−2 °C and a relative humidity of roughly 40%. They were kept on a standard 12 h/12 h light/dark cycle at an illuminance of approximately 270 lux (Osram L36W/954 FLH1) during the light phase. Pelleted standard rodent diet (Kliba, Kaiseraugst, Switzerland) and tap water were provided ad libitum.

### 2.2. Electroretinography (ERG)

Each animal underwent two ERG recordings with a minimum of 48 h in between sessions. During the first (baseline) recording session, the outer retina was probed with a white LED light; during the second session, the retina was probed with a monochromatic red-light LED of 656+/−10 nm wavelength (Figure S1). Rats were dark-adapted (for at least 12 h) overnight prior to ERG. Animals were anesthetized with a subcutaneous injection of 0.005 mg/kg fentanyl (Fentanyl-Mepha, Mepha Pharma AG, Basel, Switzerland), 0.15 mg/kg medetomidine (Dorbene, Graeub, Bern Switzerland) and 2 mg/kg midazolam (Dormicum, Roche, Basel, Switzerland); pupils were dilated with tropicamide (0.5% single dose unit (SDU); Théa) and tetracaine (1% SDU; Théa) drops were used for topic analgesia. Eyes were moisturized with 2.5% hypromellose (Goniotaire, Altaire, NX, USA) and 0.9% NaCl (B. Braun, Sempach Switzerland). Throughout anesthesia, animals remained on a heating plate at 37 °C regulated by a rectal probe (ATC 2000, World Precision Instruments). Silver-embedded thread recording electrodes were placed on the corneas; reference and ground electrodes were placed subdermally below the eyes and on the back, and above the base of the tail, respectively. All preparations prior the recordings were done in the dark using a red headlight (see Figure S1 for emission spectrum). A handheld multispecies ERG (HMsERG, Ocuscience, Henderson, USA) was used to probe outer retinal function. Full field flashes were presented in a ganzfeld dome. For the scotopic paradigm, flashes with illuminances reaching from 0.03 mcd × s/m^2^ to 25,000 mcd × s/m^2^ (log −1.5 to 4.4 mcd × s/m^2^) were presented and repeated ten times (0.03 to 0.3 mcd × s/m^2^), four times (1 to 3000 mcd × s/m^2^), and once from 10,000 mcd × s/m^2^ onwards. For testing photopic responses, light flashes ranging from 10 mcd × s/m^2^ to 25,000 mcd × s/m^2^ (log 1 to 4.4 mcd × s/m^2^) were presented (32 flashes per intensity) after a background-light adaptation of ten min at 30,000 mcd/m^2^. Stimuli durations are listed in Table S1. White light was used for light adaptation of both the baseline and the red-light ERG.

After ERG recordings, anesthesia was reversed by a subcutaneous injection of 0.12 mg/kg naloxoni hydrochloride (Naloxon, OrPha), 0.75 mg/kg atipamezole (Alzane, Graeub, Bern Switzerland) and 0.2 mg/kg flumazenil (Anexate, Roche, Basel, Switzerland).

Emission spectra and intensities of white and red ERG stimulation light as well as red headlight were measured with a CL-500A illuminance spectrophotometer (Konica Minolta).

### 2.3. Data Analysis

Both eyes were recorded simultaneously resulting in two data sets per animal. a- and b-wave amplitudes as well as implicit times were analyzed and compared using GraphPad Prism 7.04 software. Statistical significances were determined using t-tests corrected for multiple comparisons with the Holm–Sidak method (α = 0.05). Each light intensity was analyzed individually; equal variances were not assumed. Significances are indicated as follows: * *p* < 0.05, ** *p* < 0.01, *** *p* < 0.001. Results are displayed as box-and-whisker plots (boxes span the interquartile range, line within box represents the median and whiskers extend to the highest and lowest values).

Signal thresholds were determined by a comparison between signal and noise amplitudes. A signal (b-wave amplitude) was defined from noise according to the following criteria: the b-wave amplitude is significantly larger than three times the pre-stimulus noise amplitude. The noise was measured prior stimuli onset and the noise amplitude defined as the difference in μV at *t* = −20 ms and the stimulus onset (*t* = 0 ms). Stimulus to noise analysis was performed for all photopic light intensities and up to 1.5 log mcd × s/m^2^ of scotopic paradigms. Statistical significances between signal and noise (three times noise amplitude) were determined using one-sided paired t-tests.

## 3. Results

### 3.1. Far Red Light Evokes Scotopic and Photopic ERG Responses in Brown Norway and Wistar Rats

**Figure 1:**
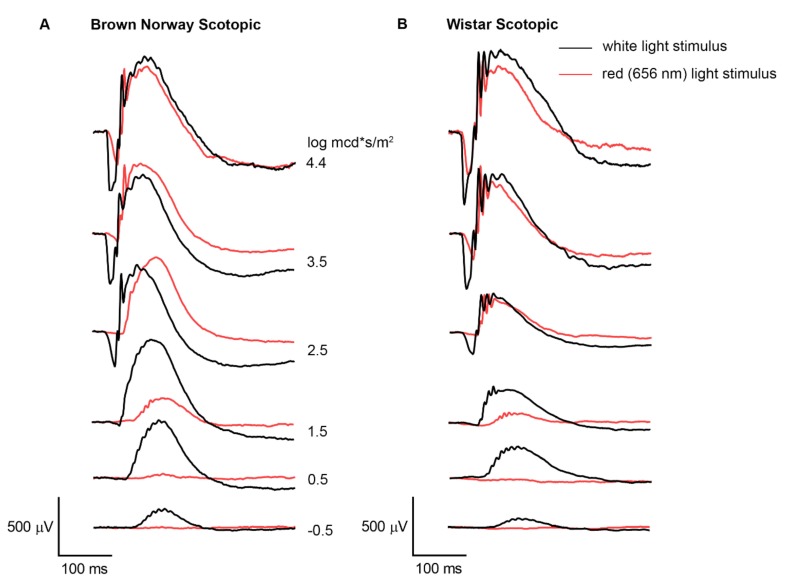

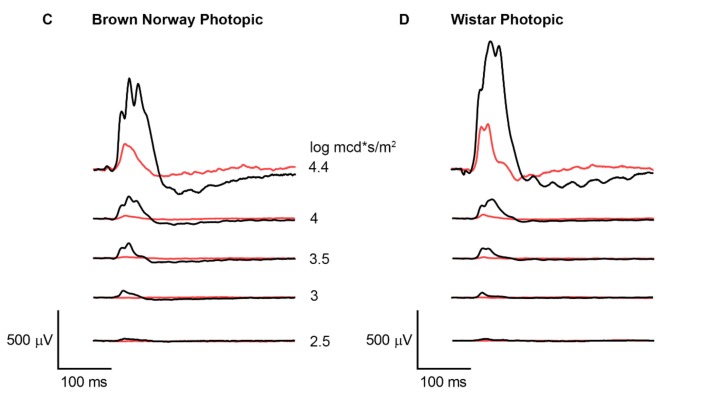
Representative electroretinogram (ERG) traces. ERG traces from a Brown Norway (**A**,**C**) and a Wistar (**B**,**D**) rat stimulated with white light (black trace) and monochromatic red light (red trace). Red light evokes a scotopic (**A,C**) and photopic (**B,D**) outer retinal response comparable in shape to white-light responses but of smaller amplitudes.

In order to assess the outer retinal response to monochromatic red light, photopic and scotopic ERG responses to red light of 656 nm were recorded and compared to baseline white light recordings. Red light of 656 nm evoked ERG responses in both pigmented Brown Norway and non-pigmented Wistar rats with kinetics comparable to the white light responses (see representative traces in Figure 1 and implicit times in Figure S2).

### 3.2. Mesopic Red-Light ERG Responses are Comparable to White-Light Responses

**Figure 2:**
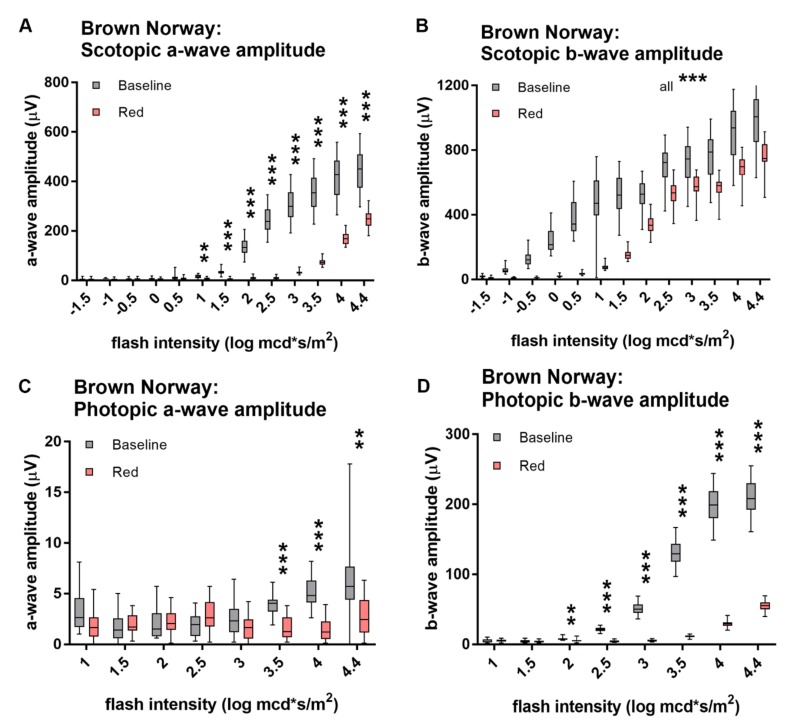
Scotopic ERG responses to white and red light. Scotopic a- (**A**,**C**) and b-wave (**B**,**D**) amplitudes of Brown Norway (**A,B**) and Wistar (**C,D**) rats stimulated with white and red light. Red-light-evoked amplitudes are significantly smaller than white-light-evoked ones, but differences in b-wave amplitudes decrease with increasing light intensities. Statistics: t-test corrected with Holm-Sidak method for multiple comparisons. ** *p* ≤ 0.01, *** *p* ≤ 0.001.

The photoreceptor-mediated scotopic a-wave in rats was smaller in amplitude if stimulated with red light compared to those induced by white light of same luminous intensity. Overall when comparing red and white light of same luminous intensity (mcd × s/m^2^), red-light-evoked b-wave amplitudes were smaller than white-light b-wave amplitudes; however, and the difference decreased with increasing light intensities (mesopic range) (Figure 2B,D) (all *p*-values are listed in Table S2). Brown Norway (Figure 2A,B) and Wistar (Figure 2C,D) rats showed comparable response patterns. Brown Norway rats showed a significant red-light response (significantly larger than three times the pre-stimulus noise amplitude) from 1 and Wistar from 3 mcd × s/m^2^ on (both *p*-values ≤ 0.0001; all *p*-values listed in Table S2).

### 3.3. Photopic Responses Evoked by Red Light are Markedly Smaller in Amplitude than Responses Evoked by White Light

**Figure 3:**
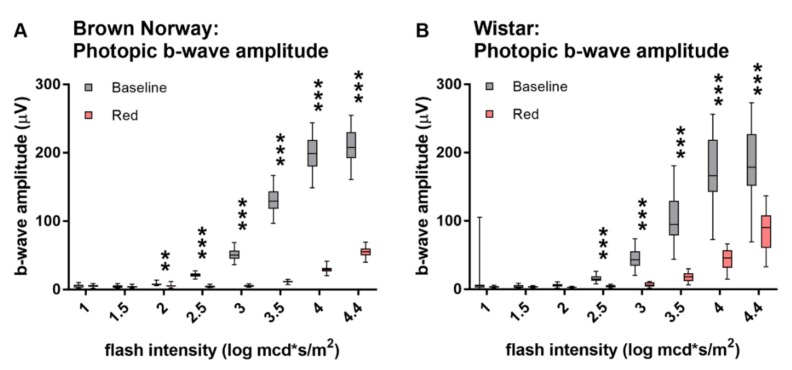
Photopic ERG responses to red and white light. Photopic b-wave responses to red and white light in Brown Norway (**A**) and Wistar (**B**) rats. Amplitudes of same intensity red light are considerably smaller than of white-light stimuli. Statistics: t-test corrected with Holm-Sidak method for multiple comparisons. ** *p* ≤ 0.01, *** *p* ≤ 0.001.

Monochromatic red light evoked a clearly discernable outer retinal response in Brown Norway and Wistar rats in photopic conditions. The b-wave amplitudes induced by red light, however, were significantly smaller than the ones induced by the white light of the same luminance (Figure 3) (*p*-values are listed in Table S2). This difference was more pronounced in Brown Norway (Figure 3A) than in Wistar (Figure 3B) rats. Red-light stimuli of 3 cd × s/m^2^ and above yielded significant ERG responses (significantly larger than three times the pre-stimulus noise amplitude) in Brown Norway Rats (*p*-values ≤ 0.0001) and stimuli of 1 cd × s/m^2^ and above yielded significant responses in Wistar rats (all *p*-values listed in Table S2).

### 3.4. Comparison of ERG Responses of Brown Norway and Wistar Rats

**Figure 4:**
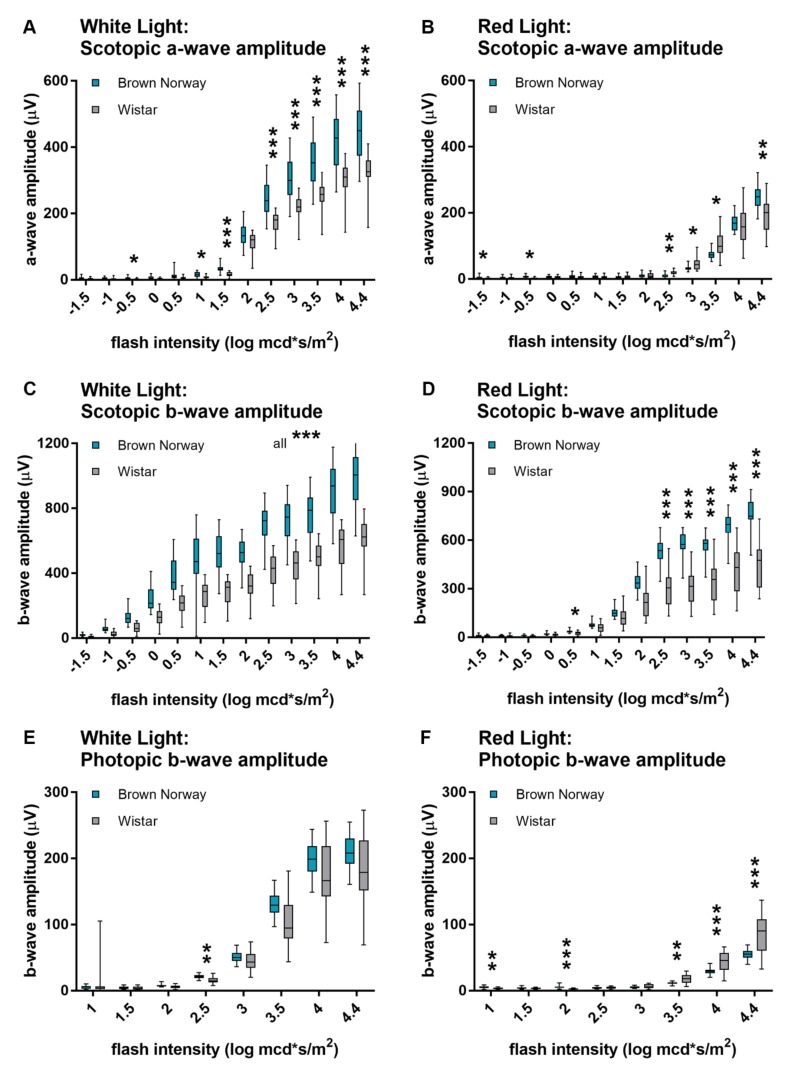
Comparison of red- and white-light induced ERG responses between Brown Norway and Wistar rats. ERG responses of white (**A**,**C**,**E**) and red (**B**,**D**,**F**) light in Brown Norway and Wistar rats. Overall, scotopic a- (**A,B**) and b-wave (**C,D**) amplitudes were larger in Brown Norway than in Wistar rats, for both red and white light. Photopic white-light responses did not differ between the two rat strains (**E**). Photopic red light evoked significantly higher b-wave amplitudes in Wistar in comparison to Brown Norway. Statistics: t-test corrected with Holm-Sidak method for multiple comparisons. * *p* ≤ 0.05, ** *p* ≤ 0.01, *** *p* ≤ 0.001.

When comparing ERG amplitudes between Brown Norway and Wistar rats (Figure 4), it became apparent that Brown Norway rats showed significantly larger scotopic white-light responses (both a- and b-wave) than Wistar rats (Figure 4A,C). However, there was no difference in the photopic white-light responses between the two strains (Figure 4E). When stimulated with red light, scotopic a-wave amplitudes were only slightly higher in Brown Norway than Wistar rats (Figure 4B), but scotopic b-wave amplitudes were considerably larger in Brown Norway rats (Figure 4D). Photopic responses to bright-red light evoked a significantly larger b-wave amplitude in Wistar compared to Brown Norway rats (Figure 4F) (all *p*-values are listed in Table S2).

### 3.5. Sex-Dependent Differences in White- and Red-Light ERG Responses

**Figure 5:**
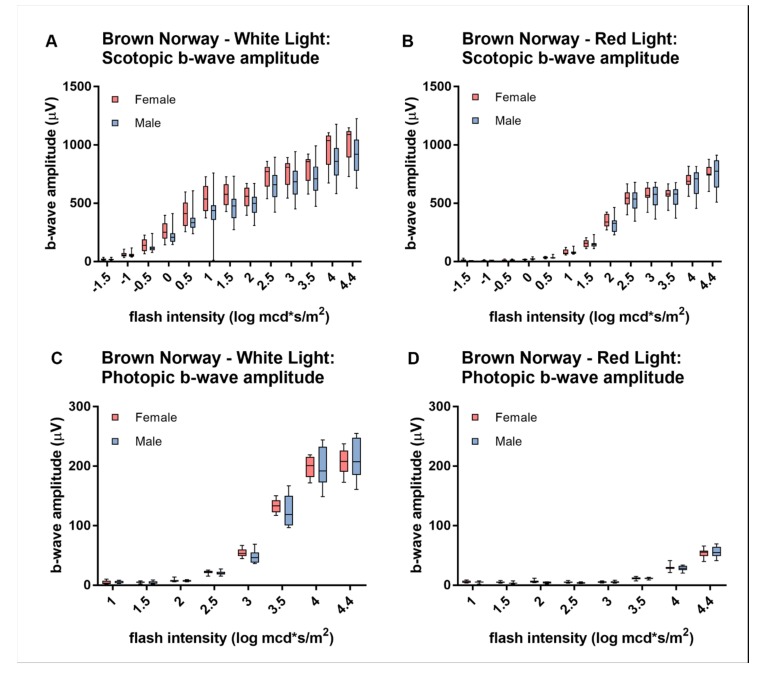
Sex differences in white- and red-light ERG responses in Brown Norway rats. There is no significant difference in the scotopic (**A**,**C**) and photopic (**B**,**D**) b-wave amplitudes between female and male Brown Norway rats, neither for white (A,B) nor for red light (**C,D**). Statistics: t-test corrected with Holm-Sidak method for multiple comparisons.

**Figure 6:**
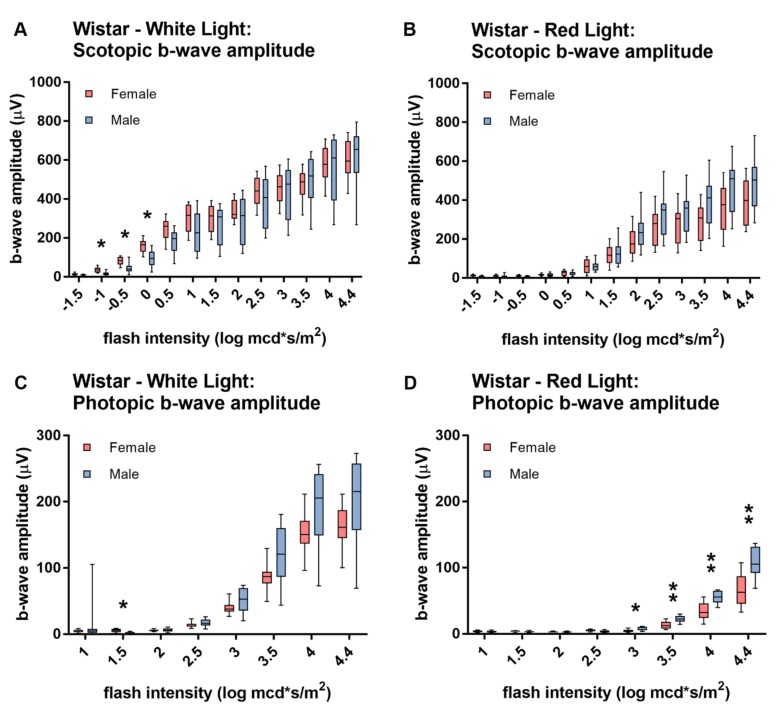
Sex-dependent differences in white- and red-light ERG responses in Wistar rats. Female Wistar rats have a slightly significantly higher scotopic white-light evoked b-wave amplitude at dim-light intensities (**A**). If stimulated with red light, there is no significant sex difference in scotopic b-wave amplitudes (**C**). There is a tendency of male Wistar rats showing a larger photopic (**B**,**D**) ON-response to bright light than female rats. This is statistically significant for red light (**D**), however not for white light (B). Statistics: t-test corrected with Holm-Sidak method for multiple comparisons. * *p* ≤ 0.05, ** *p* ≤ 0.01.

No sex-dependent differences (Figure 5) in scotopic (Figure 5A,B) and photopic (Figure 5C,D) ERG responses were detected in Brown Norway rats, neither if stimulated with white (Figure 5A,B) nor with red (Figure 5C,D) light (*p*-values in Table S2). Nevertheless, Wistar rats did reveal sex dependent differences (Figure 6). In scotopic conditions, dim-light stimuli evoke slightly larger b-wave amplitudes in female than in male rats, whereas stimuli of higher intensities do not yield a significant difference in the response amplitudes (Figure 6A). Male and female Wistar rats showed comparable scotopic responses to red light (Figure 6C). However, male rats tend to have a larger photopic b-wave amplitude if stimulated with bright light (Figure 6B,D), with a significant difference for red light (Figure 6D) (*p*-values in Table S2).

## 4. Discussion

Considering the extent to which light controls an organism’s physiology and behavior, along with its photo-damaging potential, it is crucial to precisely understand the spectral sensitivity of an organism in order to carefully control for light being a variable in experimental setups. Therefore, the aim of the current study was to analyze red-light vision in laboratory rodents. We assessed red-light evoked responses not only under photopic conditions, but also under scotopic conditions as red lights are used during the dark phase in husbandry and experiments and red light at night has been shown to substantially disrupt the circadian rhythm [19,20,21,22,23,24,25]. The electroretinogram provided a direct measure of outer retinal responses to red and white lights at different luminous intensities.

### 4.1. Interpretation of Results: Rat Red-Light Vision

Contrary to common assumptions and despite the lack of red-light-sensitive opsin (L-opsin), rods and cones of pigmented and albino rats showed a response to pure far-red light. Most importantly, the rat retina does not only respond to high intensity red light, but low intensity red light of 1 mcd × s/m^2^ (scotopic) and 1 cd × s/m^2^ (photopic) already yield significant retinal responses and thus is sensitive to red light especially in scotopic conditions. The photopic spectral sensitivity function of rats peaks at 500 nm, accompanied by a smaller peak at 361 nm, corresponding to the absorption maxima of M- and UV-cones, respectively [13]. Therefore, both, the decrease in amplitude and the higher response threshold for red-light-induced retinal activity is in accordance with the spectral sensitivity function S(λ) proposed by de Farias Rocha et al. [13]. Although red light evoked significantly smaller response amplitudes than white light of same luminous intensity, a- and b-wave amplitudes reached surprisingly large values, especially in the mesopic range. De Farias Rocha et al. observed insensitivity of the light-adapted rat retina to light of wavelengths above 620 nm [13], and laboratory guidelines suggest the use of light equaling or above 650 nm for reversed light cycle experimentation [26]. Nevertheless, our results demonstrate that the rat retina significantly responds to red light, even to wavelengths substantially longer than 620 nm. The reason for the discrepancy of the red-light-sensitivity of rats between our study and de Farias Rocha et al. is attributable to differences in experimental setups; we were using a single flash ERG paradigm on dark- and light- adapted retinas while the sensitivity function was assessed using flicker stimuli between 4 and 12 Hz light on light-adapted rats only [13]. The use of a single flash paradigm is a substantially more sensitive method than the constant flicker response to detect signals in the low sensitivity range, which is in line with the fact that amplitudes decrease with increasing flicker frequency of the same intensity. Therefore, with the single flash paradigm we were able to pick up photopic retinal responses that are below detection thresholds of a flicker ERG paradigm.

Overall, the difference in ERG amplitudes between red and white light is smaller in dark-adapted conditions in comparison to photopic conditions. Whether rods are more sensitive to red light than cones cannot be determined, as the highest signal amplitudes in the dark-adapted ERG were seen in the mesopic range where both rods and cones are functional. However, considering that animals were prepared for recordings in red light suggests that actual retinal sensitivity might be even higher than what we observed, as photoreceptors presumably were not fully dark-adapted.

Even though red light evoked surprisingly high mesopic responses, with amplitudes not much lower than amplitudes evoked by white light of the same luminous intensity, one has to consider that stimulus intensity was described using the photometric unit millicandela second per square meter (mcd × s/m^2^), as is standard for ERG. Candela is a measure of power emitted by a light source per solid angle, however as weighted by the spectral luminous efficiency function (sensitivity of the human eye to light of a certain wavelength) a rather meaningless unit for different species with different photoreceptor action spectra (e.g., rats with rods, UV and green sensitive cones). The actual power output per area (W/m^2^) of the red light was five times higher than of white light of equal luminous intensity. Thus, differences between red- and white-light response amplitudes would be even more pronounced when comparing light stimuli of same irradiances.

### 4.2. Differences of Red Light on Retinal Responses between Brown Norway and Wistar Rats

In order to get a more thorough understanding of the red-light vision of rats, we compared red (and white) light evoked stimulus amplitudes between pigmented Brown Norway and non-pigmented Wistar rats. Various studies reported larger ERG amplitudes in albino in comparison to pigmented humans, explained by higher trans-scleral illumination of the retina, less light absorption by the translucent iris, a higher fundal reflectance and greater scatter back of light in albinos [27,28,29,30]. Surprisingly, we observed significantly lower scotopic a- and b-wave amplitudes in red and white ERGs in albino rats. Photopic white light yields comparable responses in Brown Norway and Wistar rats, while photopic red-light ERG evokes significantly larger b-wave amplitudes in pigmented rats. Supporting our findings, it was reported that Wistar rats have lower scotopic a- and b-wave amplitudes compared to the genetically closely related, pigmented Long–Evans rats [31]. Whether there is a reduced number of rods in albinos versus pigmented rats, as discussed by Donatien and Jeffery [32], is unclear but could provide a possible explanation for the strain differences.

Controversially, while having comparable cone white-light responses, photopic red-light evoked responses of larger amplitudes in albino compared to pigmented rats. Melanin, the iris pigment of pigmented animals has a peak absorption in the low wavelength range (335 nm) and basically does not absorb long wavelength light above 700 nm [33]. Thus, iris pigmentation only filters out a negligible portion of red light, which may not explain our findings. Additionally, the small difference in peak spectral sensitivity in the middle wavelength range between albinos (501 nm) [31] and pigmented rats (509 nm) [34] does not provide an adequate explanation, as pigmented rats exhibit a slightly shifted sensitivity toward longer wavelengths and should, therefore, induce a larger response amplitude to red light. Hence, this interesting observation requires further investigation.

### 4.3. Sex-Dependent Differences in White- and Red-Light ERG Responses

Female and male Brown Norway rats did not differ in their outer retinal response to white and red light. Therefore, no special considerations are required when choosing the sex of experimental subjects of similar age (between 2 and 4.5 months). In Wistar rats however, we observed sex-dependent differences with males showing larger retinal ON-responses in photopic conditions and females showing larger responses to dim stimuli under scotopic conditions. While in humans, sex-related differences in structure and functions are known, research on this topic in rats is sparse. It is reported that no sex-related differences in retinal function were observed in Sprague-Dawley rats, with the exception of post-menopausal females showing larger ERG responses than age-matched males [35]. Therefore, at this point the observed sex-related differences in Wistar rats cannot be explained yet and further analysis is needed.

### 4.4. Impact of Results

Our results demonstrate that far-red light evokes a retinal response in rats. Even though ERG-measured responses are not essentially a direct measure of vision, it is safe to assume that red light is visually perceived by rats, expecting that there is no damage in the inner retina. Therefore, our data undermine that rats are not red-light blind.

ipRGCs, the non-image forming photoreceptors in the inner retina, work in conjunction with rods and cones to entrain the circadian rhythm. As melanopsin, the visual pigment of ipRGCs has a peak sensitivity to blue-green light and an insensitivity to red light [14,15,16,17], it is probable that rods and cones are (at least partially) involved in mediating red light-evoked modulation of behavior, physiology and metabolism, though this has yet to be proven experimentally.

### 4.5. Guideline for the Use of Red Light in Rodent Husbandry and Experimental Setups

Red light is commonly being used in animal experiments for two different purposes. On one hand, red light is used in reversed light cycle settings where animals need to be visually observed during their active (dark) phase. On the other hand, animals are being handled and prepared for experiments under red light when working with dark-adapted animals in ophthalmology studies (e.g., electroretinography) in order to reduce bleaching of their photopigments. In both applications, red light is used in a dark room, exactly mimicking the setting (dark-adapted) in which red light evoked the largest retinal responses (scotopic and mesopic). Therefore, it is important to consider that when using red observation lights in dark settings, the light should be as dim and of wavelengths as close to the infrared as possible. With our experiments, we demonstrate that in scotopic conditions flashes as dim as 1 mcd*s/m^2^ elicit a retinal response. Given that the headlight we used prior ERG recordings has an illuminance and irradiance of 263.7 lux or 4.27 W/m^2^ when measured at a distance of 30 cm from the light, we presume that the rat retina was not fully dark-adapted prior ERG recordings. Therefore, the retina might be even more sensitive to red light than our results suggest. As the retina is more sensitive to a flashing source (e.g., ERG stimuli) than to steady light (e.g., room lights), it is not possible to extrapolate irradiance or illuminance values that are safe to use in reversed light cycle settings based on our ERG recordings. Therefore, we advise to use monochromatic red LEDs with a peak emission of at least 660 nm and to keep them as dim as possible. For ERG recordings, it seems more suitable to use a dim, diffuse red background light source instead of a headlamp, to avoid exposing animals to variable light intensities caused by varying distances between animal and light source due to head movements. This should lead to a more uniform dark-adaptation state between animals and more consistency in the recordings between animals. The common use of headlamps (of different emission spectra and illuminances) and the resulting variability in dark-adaptation states might well explain the observed cross-institutional differences in murine ERG responses.

Generally, the same red-light source should be used at equal intensity and intervals throughout and between studies to minimize variability and control for the effect of red light on dark adaptation, endocrine levels, metabolism, and behavior, including sleep–wake behavior and locomotor activity. Furthermore, we advise to specify durations, emission spectrum and irradiance of red-light sources used when publishing studies if possible or at least the type of light source used.

## 5. Conclusions

It is of great importance to promote awareness of rodents’ ability to detect red light, which evokes surprisingly high outer retinal responses. Our evidence-based approach demonstrates the necessity of carefully considering light spectrum and illuminances for rodent husbandry to ensure optimal lighting conditions. This knowledge will help improve animal welfare by refining general housing conditions, which will have an immense 3R (reduction, refinement, replacement) impact on the ethics of animal experimentation. Additionally, we are certain that considering the variability of lighting conditions, including spectrum and intensity, in future research plans will positively influence standardization by reducing variability and enhancing the reproducibility of animal experimentation ultimately leading to a reduction in the number of animals needed.

## Supplementary Materials

The following are available online at https://www.mdpi.com/2076-2615/10/3/422/s1, Figure S1: Emission spectra of ERG stimulation light and headlight. (**A**) White LED ERG stimulation light peaks at 447 nm and 551 nm. 30,000 mcd/m^2^ (used for background adaptation) correspond to 99.3 lux and 0.274 W/m^2^. (**B**) Red LED ERG stimulation light has a peak at 656 nm. 30,000 mcd/m^2^ correspond to 92 lux and 1.32 W/m^2^. (**C**) The headlight used for ERG preparations peaks at 660 nm. At a distance of 0.3 m, the headlight has an illuminance of 263.7 lux and irradiance of 4.27 W/m^2^. Figure S2: Comparison of implicit times between white- and red-light ERG. Scotopic a-wave implicit times are higher for red than white light in the mesopic range (**A**,**B**). Scotopic b-wave implicit times show a similar pattern between white and red light, with red light showing a shift towards higher light intensities (**C**,**D**). At low light intensities, photopic b-wave implicit times are higher for red than white light, but lower at high light intensities (**E**,**F**). Statistics: t-test corrected with Holm-Sidak method for multiple comparisons. * *p* ≤ 0.05, ** *p* ≤ 0.01, *** *p* ≤ 0.0011. Table S1: Durations of ERG stimuli, Table S2: *p*-values of Figure 2, Figure 3, Figure 4, Figure 5 and Figure 6, Table S3. ERG raw data. a- and b-wave amplitude values and implicit times of all baseline and red-light ERGs and pre-stimulus noise analysis of red-light ERG.
